# LOVE HURTS: MARITAL STATUS AND CHRONIC PAIN

**DOI:** 10.1093/geroni/igae098.2877

**Published:** 2024-12-31

**Authors:** S Emily Choi, Brandon Wagner

**Affiliations:** Texas Tech University, Lubbock, Texas, United States; Texas Tech University, Lubbock, Texas, United States

## Abstract

While chronic pain is a highly prevalent and costly public health concern among older adults in the United States, longitudinal research on the social determinants of chronic pain, particularly its association with social relationships, has been limited. To address this gap, this study investigates how marital status influences later-life chronic pain with a focus on gender-specific variations. Drawing on the Health and Retirement Study (1998–2016), we estimated fixed effects logit models using an analytic sample of community-dwelling American adults aged 50 and above (N = 19,975). At baseline, women reported moderate-to-severe limiting pain (27.07%) significantly more frequently than did men (19.41%). Among men, unmarried groups, specifically the divorced/separated [OR = 0.72] and the widowed [OR = 0.55], had significantly lower odds of experiencing moderate-to-severe limiting pain compared to married individuals, controlling for demographic characteristics, socioeconomic status, health conditions and health behaviors. Among women, only the widowed had significantly lower odds of experiencing moderate-to-severe limiting pain [OR = 0.72] compared to their married counterparts. These findings suggest that though intimate social relationships may be important risk factors in shaping chronic pain in later life, they do so in perhaps surprising ways.

